# Assessment of lower urinary symptom flare with overactive bladder symptom score and International Prostate Symptom Score in patients treated with iodine-125 implant brachytherapy: long-term follow-up experience at a single institute

**DOI:** 10.1186/s12894-017-0251-1

**Published:** 2017-08-14

**Authors:** Makito Miyake, Nobumichi Tanaka, Isao Asakawa, Shunta Hori, Yosuke Morizawa, Yoshihiro Tatsumi, Yasushi Nakai, Takeshi Inoue, Satoshi Anai, Kazumasa Torimoto, Katsuya Aoki, Masatoshi Hasegawa, Tomomi Fujii, Noboru Konishi, Kiyohide Fujimoto

**Affiliations:** 1 Department of Urology, Nara Medical University, 840 Shijo-cho, Nara, 634-8522 Japan; 2 Department of Radiation Oncology, Nara Medical University, Nara, Japan; 3 Department of Pathology, Nara Medical University, Nara, Japan

**Keywords:** Prostate cancer, Brachytherapy, International Prostate Symptom Score, Overactive bladder symptom score, Biologically effective dose, PSA bounce

## Abstract

**Background:**

The aim of this study was to evaluate the combined use of the overactive bladder symptom score (OABSS) and International Prostate Symptom Score (IPSS) as an assessment tool for urinary symptom flare after iodine-125 (^125^I) implant brachytherapy. The association between urinary symptom flare and prostate-specific antigen (PSA) bounce was investigated.

**Methods:**

Changes in the IPSS and OABSS were prospectively recorded in 355 patients who underwent seed implantation. The percentage distribution of patients according to the difference between the flare peak and post-implant nadir was plotted to define significant increases in the scores. The clinicopathologic characteristics, treatment parameters, and post-implant dosimetric parameters were compared between the non-flare and flare groups. PSA bounce was defined as an elevation of ≥0.1 ng/mL or ≥0.4 ng/mL compared to the previous lowest value, followed by a decrease to a level at or below the pre-bounce value.

**Results:**

A clinically significant increase required an IPSS increase of at least 12 points and an OABSS increase of at least 6 points based on a time-course analysis of total scores and the QOL index. Assessment only by IPSS failed to detect 40 patients (11%) who had urinary symptom flare according to the OABSS. Univariate and multivariate analyses revealed that patients treated with higher biologically effective doses and those without diabetes mellitus had higher risks of urinary flare. There was no statistical correlation between the incidence and time of urinary symptom flare onset and that of a PSA bounce.

**Conclusions:**

To our knowledge, this is the first report to prove the clinical potential of the OABSS as an assessment tool for urinary symptom flare after seed implantation. Our findings showed that persistent lower urinary tract symptoms after seed implantation were attributed to storage rather than to voiding issues. We believe that assessment with the OABSS combined with the IPSS would aid in decision-making in terms of timing, selection of a treatment intervention, and assessment of the outcome.

**Electronic supplementary material:**

The online version of this article (doi:10.1186/s12894-017-0251-1) contains supplementary material, which is available to authorized users.

## Background

Low-dose rate brachytherapy using iodine-125 (^125^I) seed implantation with or without supplemental external beam radiotherapy (EBRT) is currently a standard treatment for localized prostate cancer (PCa). According to a nationwide prospective cohort study, the Japanese Prostate Cancer Outcome Study of Permanent Iodine-125 Seed Implant (J-POPS), over 25,000 patients with PCa underwent brachytherapy in Japan as of 2014 [1, 2]. A previous survey investigating the treatment distribution of primary therapy for cT1–2N0M0 PCa at our institute showed a brachytherapy rate of 38% [3]. While brachytherapy has positive long-term oncological outcomes, there are clinical concerns. These include the incidence of post-treatment adverse effects such as lower urinary tract symptoms (LUTS) and quality of life (QOL) deterioration [2, 4–11].

Worsening LUTSs are one of the most bothersome consequences of brachytherapy; however, acute symptoms tend to mitigate as the baseline is gradually restored within 1 year [4, 5]. During further follow-up, transient relapse of LUTS is observed in some patients, which Cesaretti et al. termed a “urinary symptom flare” [12]. Since then, predictive factors of urinary symptom flares have been explored in large cohorts of patients undergoing brachytherapy [4, 8–11]. In previous reports, chronological changes on International Prostate Symptom Score (IPSS) questionnaires were used to evaluate the urinary symptom flare. The results showed that urinary symptom flare incidence was associated with erectile dysfunction, higher baseline IPSS, maximal post-implant IPSS, age, the biologically effective dose (BED), and implementation of supplementary EBRT [4, 9, 11].

The IPSS has been predominantly utilized as a tool for defining urinary symptom flares, which are termed IPSS flares [4, 8–12]. Bothersome symptoms after radiotherapy seem to mainly be characterized by storage urinary characteristics including frequency, urgency, and nocturia [13]. The overactive bladder symptom score (OABSS) was developed in 2006 as an evaluation tool for patients with overactive bladder syndrome (OAB) and has been validated in Japanese as well as in other populations [14–16]. The OABSS evaluates relevant symptoms with only 4 questions that cover daytime frequency, nocturia, urgency, and urge incontinence. This questionnaire is simple and quick, and it agrees with corresponding diary variables [17] and treatment-related improvement with anticholinergic use [18]. Therefore, the OABSS may be beneficial for evaluating urinary symptom flares after brachytherapy.

To date, no reported studies have investigated urinary symptom flare after ^125^I brachytherapy in a single cohort by using both the IPSS and the OABSS. In this prospective study, we focused on the predictive factors for urinary symptom flare based on the IPSS and OABSS questionnaires. In addition, we investigated the association between urinary symptom flare and the incidence of prostate-specific antigen (PSA) bounce because research on this issue is limited.

## Methods

### Patients and data collection

Between April 2004 and September 2010, 355 consecutive patients underwent ^125^I brachytherapy for localized PCa in the Nara Medical University Hospital. The clinicopathologic characteristics of the patients, the treatments, and the BED are listed in Table 1. The median age was 71 years (range, 48–83). The median initial PSA was 7.11 ng/mL (range, 3.10–32.2). Two pathologists (T. Fujii and N. Konishi) with expertise in Pca diagnosis reviewed the Gleason scores of all biopsy specimens. Tumor stages were identified according to the 2002 Union for International Cancer Control classification. Patients were stratified according to the D’Amico risk classification [19]. The baseline (BL) urinary function was prospectively determined by the IPSS, IPSS-QOL index, OABSS, Sexual Health Inventory for Men (SHIM), and a voiding study before implantation and during the post-implant follow-ups that were conducted at 1, 3, 6, 12, 24, 36, 48, and 60 months after implantation. The voiding symptoms-related IPSS (V-IPSS; the sum of questions 1, 3, 5, and 6) and storage symptoms-related IPSS (S-IPSS; the sum of questions 2, 4, and 7) subscores were calculated separately [13]. PSA level was monitored 1, 3, 6, 12, 18, 24, 30, 36, 48, and 60 months after implantation. In the present study, PSA bounce was defined as an increase of at least 0.1 ng/mL or 0.4 ng/mL (two different cutoffs) more than the previous lowest value (excluding the 1 month PSA value), followed by a decrease to a level at or below the pre-bounce value [20].

**Table 1:** Characteristics of 355 patients

Variables	Total (*n* = 355)	%
Age at brachyterapy (years) ^a^	71 (48–83)	
Initial PSA (ng/mL) ^a^	7.11 (3.10–32.2)	
Clinical T category		
T1c	197	55%
T2a	128	36%
T2b or T2c	30	8%
D’Amico risk classification		
Low	153	43%
Intermidiate	166	47%
High	36	10%
Gleason sum		
6	206	58%
7	131	37%
8 or 9	18	5%
Hypertention		
No	243	68%
Yes	112	32%
Diabetis		
No	318	90%
Yes	37	10%
Pre-use of alpha-1 adrenoceptor antagonist		
No	304	86%
Yes	51	14%
Baseline IPSS (0 to 35)		
Continuous ^a^	7 (0–33)	
1 to 7	201	57%
8 to 19	130	37%
20 to 35	23	6%
Baseline OABSS (0 to 15)		
Continuous ^a^	3 (0–13)	
0 to 5	289	81%
6 to 11	59	17%
12 to 15	4	1%
Prostate volume at diagnosis (mL) ^a^	24.2 (7.8–59.9)	
Prostate volume at implant (mL) ^a^	25.7 (7.8–61.9)	
Supplementary EBRT		
No	247	70%
Yes	108	30%
Combined ADT		
No	227	64%
Yes	128	36%
No of needles ^a^	23 (14–36)	
No of seeds ^a^	60 (30–95)	
BED (Gy2)	194.8 (120.3–253.2)	

*PSA* prostate-specific antigen, *IPSS* International Prostate Symptom Score, *OABSS* overactive bladder symptom score, *SD* standard deviation, *EBRT* external beam radiotherapy, *ADT* androgen deprivation therapy, *BED* biologically effective dose

^a^ expressed by medians and ranges

### Definition of urinary symptom flare

Patients in the study and in previous reports [5] experienced a transient elevation of urinary symptom scores after implantation. After the peak, the scores subsequently returned to the BL. The lowest score after the peak is termed the “urinary symptom nadir.” A subgroup of patients experienced a relapse in negative urinary symptoms without any bacterial urinary tract infections, which is called a “urinary symptom flare.” Previous studies defined the IPSS of 5 or 8 points greater than the post-implant nadir of the IPSS as urinary symptom flare. However, the optimized cutoff point has not been defined yet. To define the significant increase in the IPSS and OABSS in a Japanese cohort, we plotted the percentage distribution of patients according to the difference between the flare peak and post-implant nadir as previously reported [9, 12]. Two cutoff points for the difference between the flare peak and nadir were established, so that approximately 25 and 50% of patients, respectively, were classified as experiencing urinary symptom flare. To examine significant factors predicting the occurrence of this condition, clinicopathologic variables and post-implant dosimetric parameters were evaluated with univariate and multivariate analyses.

### Procedure of the seed implantation, EBRT, and post-implant dosimetry

We performed the implantation procedure as previously described [20, 21]. The prescribed radiation dose was 145 or 160 Gy in the implantation monotherapy group and 110 Gy of radiation combined with 45 Gy of EBRT administered in 25 fractions in the combined group [21]. Of the 355 patients, 108 (30%) were treated with combined androgen depletion therapy (ADT) in which luteinizing hormone-releasing hormone agonist, anti-androgen, or a combination of the two was used. An experienced radiation oncologist (I. Asakawa) performed a computed tomography scan about 1 month after implantation to obtain the post-implant dosimetric parameters. The BED was calculated with the formulas reported by Stock et al., in which the prostate D90, EBRT dose, and α/β ratio of 2 (Gy2) for the effect-specific parameter were taken into account [22].

### Statistical analysis

We evaluated chronological changes by plotting each IPSS and OABSS in line graphs where scores were expressed as the mean ± standard deviation (SD). The Mann-Whitney *U*-test, chi-square test, and Fisher’s exact test were used to analyze the clinicopathologic variables. The independent association was evaluated using the odds ratio (OR) and a 95% confidence interval (CI) derived from standard logistic regression methods. For multivariate analysis, variables were selected on the condition that the *P* value was less than 0.1 in the univariate analysis. The interrelationship between the time of the urinary symptom flare and the time of the PSA bounce was examined using Spearman’s correlation. IBM SPSS version 21 (SPSS Inc., Chicago, IL, USA) and Prism software 5.00 (GraphPad Software, San Diego, CA, USA) were utilized for statistical analyses and data plotting, respectively. A *P* value of <0.05 was considered statistically significant.

## Results

### Time-course changes in the IPSS, OABSS, and subscores after seed implantation

**Fig. 1:**
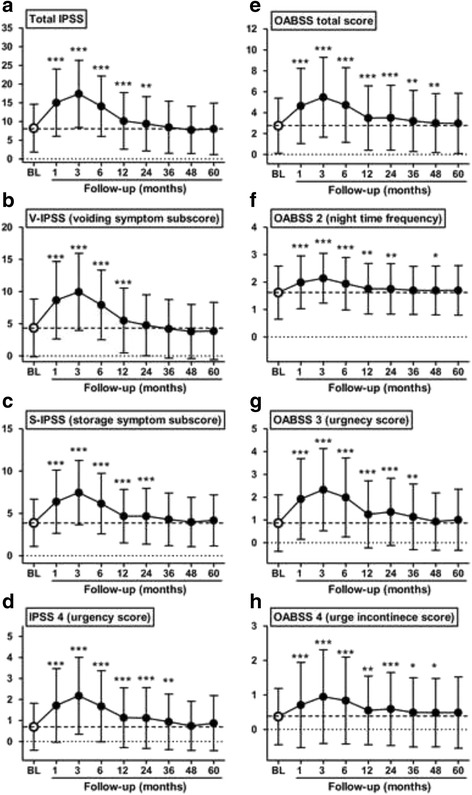
Changes in representative parameters for lower urinary symptoms during follow-up after seed implant. IPSS (**a−d**) and OABSS (**e−h**) questionnaires were used for evaluation. Scores at each time point (the baseline and 1, 3, 6, 12, 24, 36, 48, and 60 months after implant) were compared with the baseline scores with the Mann-Whitney *U*-test. Data are expressed by means and standard deviations. Changes in other parameters are shown in Additional file 1: Figure S1. BL, the baseline; * *P* < 0.05, ** *P* < 0.01, *** *P* < 0.001

The clinicopathologic characteristics, treatment parameters, and BED are listed in Table 1. The median follow-up after implantation was 72 months (range, 2–126). The changes in the IPSS and OABSS from BL to 60 months after implantation were plotted on line graphs (Fig. 1a-h and Additional file 1: Figure S1). For all analyzed data, the worst symptom scores were observed 3 months after implantation; however, most scores decreased with time. Although the V-IPSS returned to the BL after 24 months (Fig. 1b), the S-IPSS did not (Fig. 1c), resulting in an elevated total IPSS at 24 months (Fig. 1a). The worsening of the urgency score persisted most among the 7 scores of IPSS questionnaire (Fig. 1d and Additional file 1: Figure S1). While the total IPSS returned to the BL after 36 months, the heightened total OABSS, OABSS 2 (night time frequency), and OABSS 3 (urge incontinence) lasted for over 48 months (Fig. 1e, f, and h). OABSS 3 (urgency score) returned to the BL after 48 months, similar to the results of the IPSS 4 (urgency score) (Fig. 1d and g). These findings support the idea that persistent LUTS after implantation are attributed to persistent storage rather than voiding symptoms.

### Analysis and definition of the urinary symptom flare in IPSS and OABSS

**Fig. 2:**
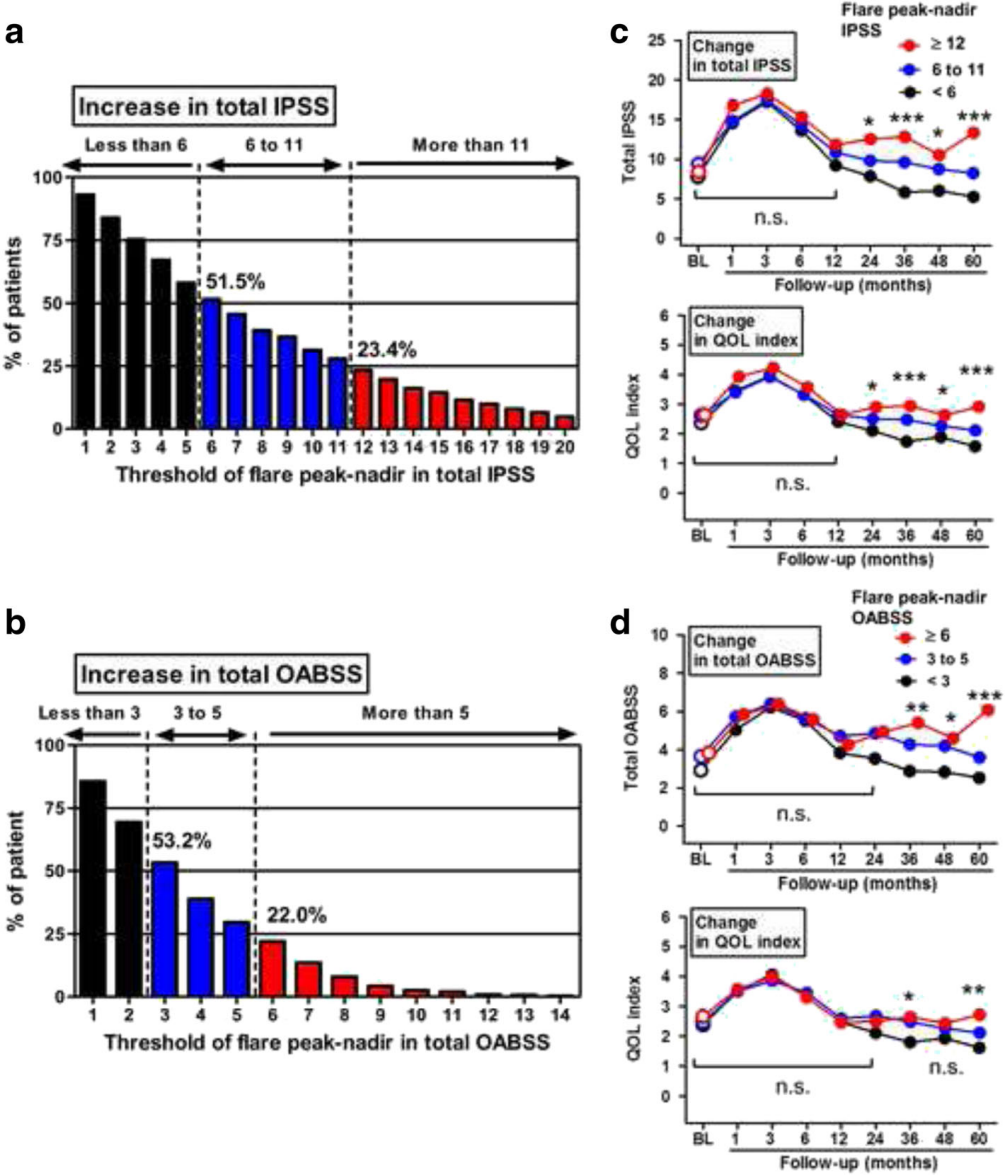
Percentage distribution of patients for IPSS and OABSS urinary symptom flare after seed implant. Cutoffs for the difference between the flare peak and nadir were established. Two vertical dashed lines delineate approximately 25% and 50% of patients who experienced the urinary symptom flare assessed by the IPSS (**a**) and the OABSS (**b**). Time-course changes in total IPSS (**c**) and QOL (**d**) index scores during follow-up are plotted according to the flare peak-nadir of total IPSS (< 6, 6–10, and ≥ 12). Time-course changes in total OABSS and QOL index scores during follow-up are plotted according to the flare peak-nadir of total OABSS (< 3, 3–5, and ≥ 5). Data are plotted by means. Scores of groups with less than 6 and 6 to 10 point increases and the group with at least a 12-point increase are compared in each time point by the Mann-Whitney *U*-test. n.s, not significant; BL, baseline; * *P* < 0.05, ** *P* < 0.01, *** *P* < 0.001

The second increase in IPSS and OABSS scores and the flare incidence are shown in Fig.s 2a and b, respectively. The mean absolute increases ± the SD of the IPSS and the OABSS were 4.6 ± 4.0 points (median 4; range 0–20) and 3.3 ± 2.7 points (median 3; range 0–14), respectively. In the analysis of IPSS flare, when the cutoff values of an IPSS increase were set at ≥ 6 points and ≥ 12 points, the flare incidences were 51.5 and 23.4%, respectively (Fig. 2a). The time-course line graphs for total IPSS and the QOL index of the three groups divided according to IPSS increases are plotted in Fig. 2c. The patients with an IPSS increase of >11 points had persistent decrease of both types of IPSS even 60 months after implantation, while the other two groups (an IPSS increase of ≤ 11points) returned to the BL with time. The time-course change in the QOL index was almost parallel to that of the IPSS (Fig. 2c, lower panel). The same analysis was performed for the OABSS with similar results (Fig. 2b and d). When the cutoff values of an OABSS increase were set at ≥ 3 and ≥ 6 points, the flare incidence was 51.5 and 23.4%, respectively (Fig. 2a). The distribution of patients based on the time until urinary symptom flare is listed according to the two cutoff values in the IPSS and the OABSS (Additional file 2: Table S1). Approximately 30% of patients developed the flare between the second and third year, while 15–20% had a flare during the fifth year.

Based on these findings, those who had an increase of 12 points or more in the IPSS and an increase of 6 points or more from the nadir in an OABSS were thought to have a clinically significant flare in urinary symptoms after seed implantation. For further analyses, higher cutoff values in the IPSS and OABSS were used to define the urinary symptom flare and divide the patients into a “flare group” and a “non-flare group.”

### Correlation between IPSS flare and OABSS flare

**Fig. 3:**
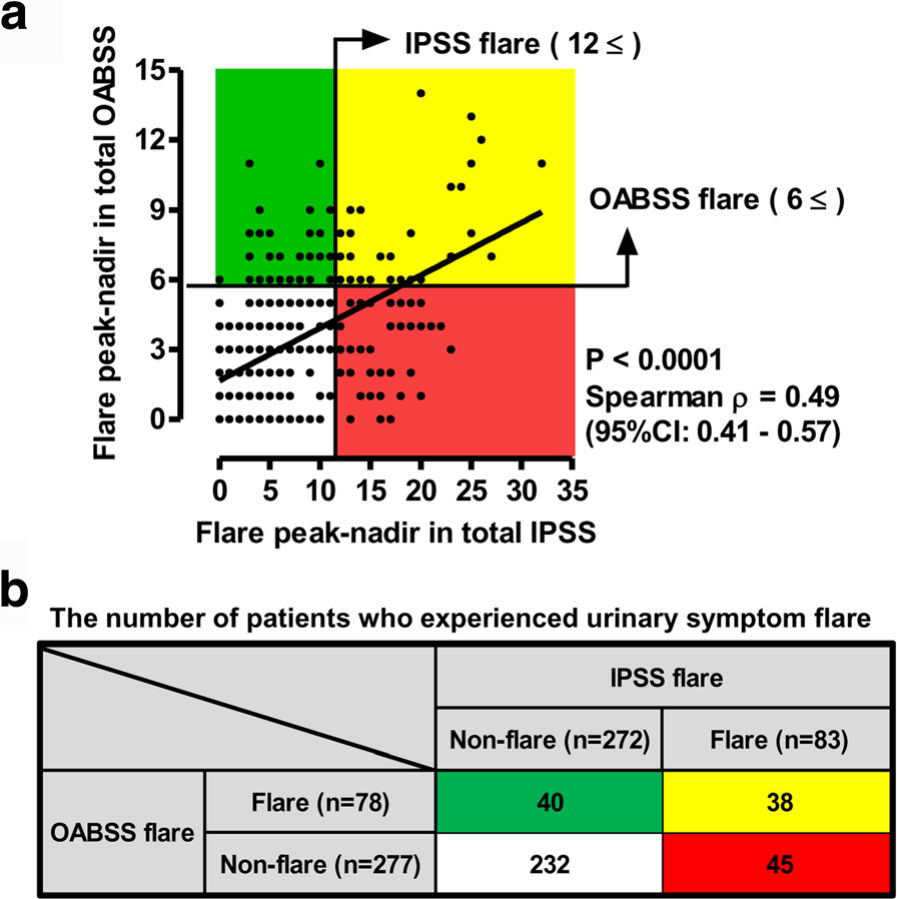
The interrelationship between IPSS flare and OABSS flare after seed implantation. **a** The points of increase (peak subtracted by nadir) for the total IPSS and total OABSS are shown. The correlation was examined using Spearman’s correlation. Spearman’s ρ and the 95% confidence interval are shown in the figures. The half-tone area indicates patients experiencing urinary symptom flare, which is defined as having at least a 12-point increase in total IPSS and a 6-point increase in total OABSS. **b** Numbers of patients who experienced IPSS flare and OABSS flare

To evaluate the correlation between IPSS flare and OABSS flare, the number of additional points (peak subtracted by nadir) in total IPSS and total OABSS were plotted and examined using Spearman’s correlation (Fig. 3a). There was a moderate correlation between the increased points of total IPSS and total OABSS (ρ = 0.49, *P* < 0.0001). Of 355 patients, 38 (11%) showed both IPSS flare and OABSS flare, whereas 75 (21%) showed either IPSS flare or OABSS flare (Fig. 3b). A high concordance rate was observed for the assessment of urinary symptom flare by IPSS and OABSS (76%, 270 out of 355 patients). However, our findings revealed that assessment solely with the IPSS questionnaire failed to detect 40 patients (11%) with urinary symptom flare whose predominant complaint was likely to be storage symptoms after seed implantation. In contrast, we found that assessment solely with the OABSS questionnaire failed to detect 45 patients (13%) with urinary symptom flare. Our findings revealed that a certain percentage of people will be independently discovered as flare on OABSS and IPSS, without overlap. The OABSS can be used as a validated patient reported outcome to assess LUTS after brachytherapy in addition to the IPSS.

### Comparison of clinicopathologic parameters between the flare group and non-flare group

**Fig. 4:**
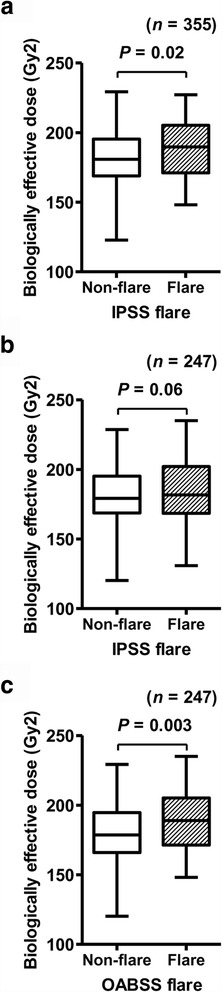
The association between biologically effective dose (BED) and urinary symptom flare. The BED calculated from post-implant dosimetry is depicted by Tukey box plots. Horizontal lines within boxes indicate median levels. **a**, Comparison of BED between the IPSS non-flare group (*n* = 272) and flare group (*n* = 83) in the 355 total patients. **b** and **c**, Comparison of BED between the IPSS non-flare group (*n* = 190) and flare group (*n* = 57) in the 247 patients who did not undergo supplementary EBRT (**b**) and between the OABSS non-flare group (*n* = 193) and flare group (*n* = 54) (**c**). Significance was tested using the Mann-Whitney *U*-test

Of 355 patients, 83 (23.4%) and 78 (22.0%) were categorized as the “flare group” based on the point increase in the IPSS and the OABSS, respectively. The clinicopathologic characteristics, treatment parameters, and post-implant dosimetric parameters were compared between the non-flare group and flare group (Additional file 3: Table S2). Among the studied variables, a higher BED (*P* = 0.02) was associated with IPSS flare incidence (Fig. 4a). Patients without diabetes mellitus (DM) were more susceptible to an IPSS flare compared to those with DM (*P* = 0.02). Patients with higher Gleason sums had a tendency of having higher incidences of IPSS flares (*P* = 0.09). Independent predictive parameters for IPSS flares were then identified. Based on *P* values of less than 0.1 on the univariate analysis, BED, DM, and the Gleason sum were included in the multivariate analysis. The optimal cut-off values of BED were determined using a dichotomous test. Multivariate logistic regression analysis identified BED (< 200 *v.* ≥ 200; OR = 1.81; *P* = 0.02) and DM (no *v.* yes; OR = 0.26; *P* = 0.03) as being independently associated with the incidence of IPSS flare (Table 2). No significantly different variable was detected in the OABSS non-flare and flare groups, older patients showed a trend of experiencing an OABSS flare more frequently (*P* = 0.06, Additional file 3: Table S2). Contrary to a previous report [4], urinary symptom flare was not associated with baseline erectile dysfunction, as measured with SHIM.

**Table 2:** Multivariate analysis for IPSS flare

Variables	OR	95% CI	*P* value
Gleason sum			
6	1		
7/8/9	1.43	0.84–2.43	0.18
Diabetes mellitus			
No	1		
Yes	0.26	0.07–0.87	0.03
BED (Gy2)			
< 200	1		
200 ≤	1.81	1.10–2.98	0.02

*OR* odds ratio, *CI* confidence interval, *BED* biologically effective dose

To exclude the effect of supplementary EBRT, a subgroup analysis of the 247 patients who were not administered supplementary EBRT was performed. The clinicopathologic characteristics, treatment parameters, and post-implant dosimetric parameters are listed in Additional file 4: Table S3. Of 247 patients, 57 (23.1%) and 54 (21.8%) were determined to be the “flare group” according to an increase in the IPSS and the OABSS, respectively. The clinicopathologic characteristics, treatment parameters, and post-implant dosimetric parameters were compared between the non-flare group and flare group (Additional file 5: Table S4). Patients with higher BED (*P* = 0.06, Fig. 4b), D90 (*P* = 0.06), and V100 (*P* = 0.08) tended to have higher incidences of IPSS flare. Among the studied variables, only higher BED (*P* = 0.003, Fig. 4c) and higher D90 (*P* = 0.001) were associated with OABSS flare incidence (Additional file 5: Table S4).

### The association between the urinary symptom flare and PSA bounce

**Fig. 5:**
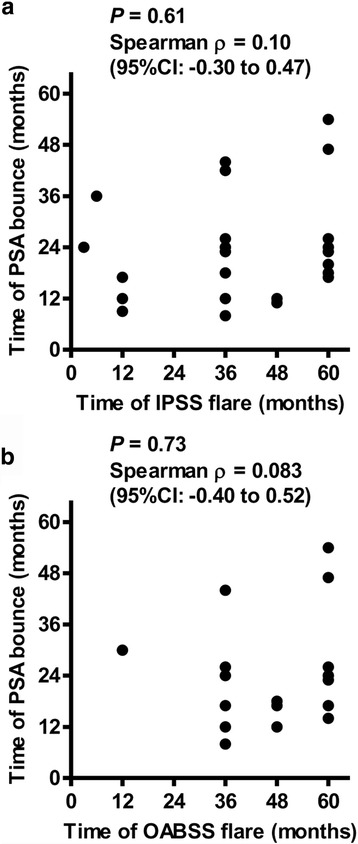
The interrelationship between time of the urinary symptom flare and time of PSA bounce. The patients who experienced both a urinary symptom flare and a PSA bounce were included. There were 27 patients with an IPSS flare and 19 with an OABSS flare. The correlation was examined using Spearman’s correlation. Spearman ρ values and the 95% confidence intervals are shown in the figures

A temporary increase in the PSA level after the post-implant nadir is called PSA bounce, and it occurs during the first 12 to 36 months after radiotherapy in the majority of cases [23]. We hypothesized that there was a clinical association between two phenomena, the urinary symptom flare and PSA bounce. As testosterone recovery and ADT affect the PSA level, patients treated with neoadjuvant and/or adjuvant ADT (*n* = 128) were excluded in order to determine the frequency of true PSA bounce. Of 355 patients, 227 (64%) were included in the analysis. A PSA bounce of 0.1 ng/mL or more was observed in 101 (44%) of the 227 patients. The mean ± SD and the median time until the PSA bounce were 23.5 ± 12.4 months and 20.0 months (range: 7−71), respectively. The mean height and duration of the PSA bounce were 0.45 ng/mL (median: 0.23 ng/mL) and 12.3 months (median: 7.0 months), respectively. On the analysis of IPSS flare, 27 (26.7%) of the 101 patients with PSA bounce experienced the flare, while 32 (25.4%) of the 126 patients without PSA bounce (*P* = 0.47, Additional file 3: Table S2) had a flare. The same analysis was performed for OABSS flare, revealing that 19 (18.8%) of the 101 patients with PSA bounce experienced the flare as compared to 32 (25.4%) of the 126 patients without PSA bounce (*P* = 0.15, Additional file 6: Table S5). Next, we performed a correlation analysis between the time of the urinary symptom flare and the time of the PSA bounce in the patients who experienced both phenomena (Fig. 5). Neither the time of the IPSS flare (*n* = 27) nor the time until the OABSS flare (*n* = 19) correlated with the time of PSA bounce. The results demonstrated no significant clinical association between a urinary symptom flare and PSA bounce. When PSA bounce of 0.4 ng/mL or more was defined as the cutoff, PSA bounce was observed in 29 (12.7%) of the 227 patients. The mean ± SD and the median time to the PSA bounce were 20.7 ± 8.0 months and 19.0 months (range: 8−48), respectively. The mean height and duration of the PSA bounce were 1.08 ng/mL (median: 0.84 ng/mL) and 13.0 months (median: 12.0 months), respectively. When the same analysis was performed, no significant clinical association was found between a urinary symptom flare and PSA bounce of 0.4 ng/mL or more (Additional file 7: Table S6).

## Discussion

The initial worsening of LUTS after implantation has been thoroughly studied by several groups [2, 5, 8, 10, 11]. We previously reported that the total IPSS and QOL index peak approximately 3 months after implantation and gradually return to the BL scores after 12 months [5]. The etiology of worsening LUTS revealed that the peak IPSS after seed implantation was associated with the total amount implanted and the dose delivered to the prostatic gland, bladder, and urethra [24]. The recurrent LUTS exacerbation after a variable asymptomatic period occurred between 6 and 60 months after seed implantation [12], which is called “urinary symptom flare.” The IPSS flare has been utilized predominantly as a tool for determining urinary symptom flare [4, 8–12]. A previous study evaluated the spectrum of pathophysiology underlying persistent LUTS after seed implantation [25]. In their cohort, 79% of the patients had overactive symptoms, and 71% had urinary incontinence, while only 44% had obstructive symptoms. A urodynamic study revealed that men undergoing seed implantation had a much higher incidence of detrusor overactivity. Many urologists and general practitioners currently use the IPSS to evaluate the severity of LUTS, decide upon an intervention, and assess the treatment outcome. Total IPSS was reported as unreliable for correctly diagnosing bladder outlet obstruction and OAB [26]. Additionally, voiding symptoms and storage symptoms do not always directly reflect those dysfunctions [27]. Since persistent LUTS is largely characterized by storage symptoms, it may be reasonable to use the OABSS to evaluate changes in LUTS after implantation. In the present study, the OABSS as well as the IPSS were accurate, useful, and sensitive to changes during a follow-up survey for patients with localized PCa after seed implantation (Fig. 2b).

Although some reports regarding urinary symptoms after seed implantation have been published, an accepted definition of urinary flare does not exist. An increase in urinary symptom score seems to be useful and practical in clinical settings and academic research. In the first detailed evaluation by Cesaretti et al., when an increase in total IPSS of ≥ 5 points was defined as a urinary flare, 36% of patients were determined to experience a flare [12]. In the report by Keyes et al., two different definitions of flare, an increase in total IPSS of ≥ 5 and ≥ 8 points, were determined, and significant predictive factors for flare were explored separately [9]. In this study, an increase in total IPSS of ≥ 12 points and of ≥ 6 points in the OABSS were determined to be clinically significant (Fig. 2). The definition used in this study is thought to be reasonable because it qualifies patients who experience long-lasting LUTS and lower QOL (Fig. 2c and d).

With our definition, approximately 25% of patients were determined to experience urinary symptom flare after the nadir. A multivariate analysis of the possible predictors for the incidence of urinary symptom flare revealed that patients treated by higher BED stratified to <200 Gy2 and ≥200 Gy2 and patients without DM had higher risks of urinary flare (Table 2). A recent paper from Japan demonstrated that high BED was associated with the incidence of IPSS flare (defined as an increase in total IPSS of ≥ 5 points) and urinary toxicity of CTCAE grade 2 or higher, but no significant association was found between BED and the first IPSS resolution [11]. In the present study, the presence of DM was associated with a lower risk of having an IPSS flare, which was a controversial result [9, 11]. One possible explanation is that the urinary symptom flare is masked by the generally worse baseline urinary function and symptoms in those with DM as compared to those without the condition [28]. Neuropathy, which is caused by ischemic change, is known to be a DM-related comorbidity. Insensitivity to inflammation and irritability, which is usually induced by radiotherapy, may be another explanation.

With regards to the PSA bounce after radiotherapy, the predictors of bounce, features that distinguish benign bounce and biochemical failure, and the prognostic impact of bounce have been well studied [29–32]. However, the detailed mechanism underlying PSA bounce remains unclear, as does the mechanism behind a urinary symptom flare. We decided to investigate the association between urinary symptom flare and PSA bounce after implantation because the data are extremely limited [12]. In our cohort, PSA bounce was observed at a similar rate of approximately 20% in the non-flare and flare groups, showing no significant difference (Additional file 2: Table S2). An additional correlation analysis revealed that the two phenomena did not occur with similar timing. Our findings and the data reported by Cesaretti et al. strongly suggested that the etiologies of bounce and urinary flare are distinct.

Limitations of this study include the relatively small sample size, which lowers the ability to obtain significant results and identify other predictors of urinary symptom flare. This was a single-institution nonrandomized study. Moreover, assessment with the IPSS and OABSS questionnaires was only conducted once a year, starting from the second year after seed implantation. More frequent assessments, such as once every 3 to 6 months, may improve the findings.

## Conclusions

To our knowledge, the present study is the first to demonstrate the clinical potential of the OABSS as an assessment tool for urinary symptom flare after seed implantation. We also investigated the predictors of IPSS and OABSS flares and the correlation between PSA bounce and urinary symptom flare. Although this single institution study, with only one surgeon, may not be representative of the experiences in the wider community, the data reflect the current status of LUTS management after implantation. Symptom flare is common and occurs in many patients within 5 years. A future prospective multi-center clinical trial will be needed to develop strategies for employing medications such as alpha-1 adrenoceptor antagonist, anti-cholinergic drugs, and phosphodiesterase type 5 inhibitors such as tadarafil (Cialis, Adcirca) to palliate bothersome symptoms. We believe that combined assessment with the OABSS and IPSS is useful when faced with the decision-making process, as it helps with both the timing and selection of treatment intervention, as well as with tracking of the outcome.

## Additional files

Additional file 1: Figure S1. Changes in parameters for lower urinary symptoms during follow-up after seed implantation. Scores, which are not shown in Fig. 1, were compared with the baseline scores with the Mann-Whitney *U*-test. Data are expressed as means and standard deviations. BL, the baseline; * *P* < 0.05, ** *P* < 0.01, *** *P* < 0.001. (TIFF 5208 kb)

Additional file 2: Table S1. Time to urinary symptom flare. (DOCX 35 kb)

Additional file 3: Table S2. Comparison of clinicopathologic parameters by IPSS flare and OABSS flare in the 355 patients. (DOCX 45 kb)

Additional file 4: Table S3. Characteristics of 247 patients without supplementary EBRT. (DOCX 37 kb)

Additional file 5: Table S4. Comparison by IPSS flare and OABSS flare in the 247 patients without supplementary EBRT. (DOCX 47 kb)

Additional file 6: Table S5. Comparison of PSA bounce and urinary symptom flare in patients without androgen deprivation therapy. PSA bounce was defined as an elevation of ≥0.1 ng/mL compared to the previous lowest value, followed by a decrease to a level at or below the pre-bounce value. (DOCX 35 kb)

Additional file 7: Table S6. Comparison of PSA bounce and urinary symptom flare in patients without androgen deprivation therapy. PSA bounce was defined as an elevation of ≥0.4 ng/mL compared to the previous lowest value, followed by a decrease to a level at or below the pre-bounce value. (DOCX 35 kb)

## Abbreviations

^125^I: Iodine-125
ADT: Androgen-deprivation therapy
BED: Biologically effective dose
BL: Baseline
CI: Confidence interval
CTCAE: Common Terminology Criteria for Adverse Events
D90: Minimal does (Gy) received by 90% of the prostate gland
DM: Diabetes mellitus
EBRT: External beam radiotherapy
IPSS: International Prostate Symptom Score
J-POPS: Japanese Prostate Cancer Outcome Study
LUTS: Lower urinary tract symptoms
OAB: Overactive bladder
OABSS: Overactive bladder symptom score
OR: Odds ratio
PCa: Prostate cancer
PSA: Prostate-specific antigen
QOL: Quality of life
RP: Radical prostatectomy
SD: Standard deviation
SHIM: Sexual Health Inventory For Men
S-IPSS: Storage symptoms-related IPSS
V-IPSS: Voiding symptoms-related IPSS
