# Supplementary figures and images for: Assessment of lower urinary symptom flare with overactive bladder symptom score and International Prostate Symptom Score in patients treated with iodine-125 implant brachytherapy: long-term follow-up experience at a single institute
Source: BMC Urol. 2017 Aug 14;17:62. doi: 10.1186/s12894-017-0251-1 (PMC5556596; doi:10.1186/s12894-017-0251-1)

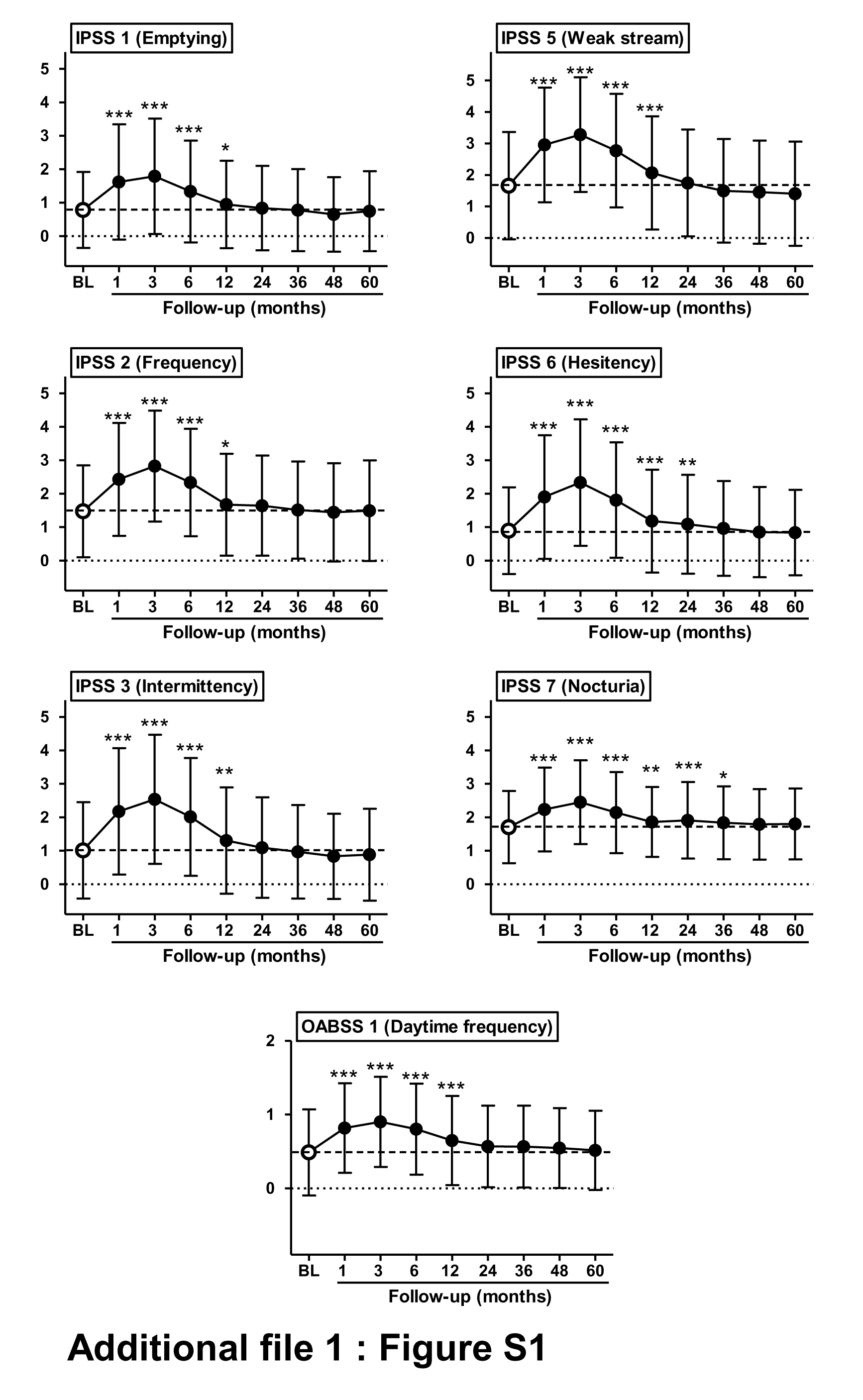

Supplement: Supplementary file 1 — Changes in parameters for lower urinary symptoms during follow-up after seed implantation. Scores, which are not shown in Fig. 1, were compared with the baseline scores with the Mann-Whitney U-test. Data are expressed as means and standard deviations. BL, the baseline; * P < 0.05, ** P < 0.01, *** P < 0.001. (TIFF 5208 kb) [file 12894_2017_251_MOESM1_ESM.tif]
