# Supplementary material for: Assessment of lower urinary symptom flare with overactive bladder symptom score and International Prostate Symptom Score in patients treated with iodine-125 implant brachytherapy: long-term follow-up experience at a single institute
Source: BMC Urol. 2017 Aug 14;17:62. doi: 10.1186/s12894-017-0251-1 (PMC5556596; doi:10.1186/s12894-017-0251-1)
Supplement: Supplementary file 2 — Time to urinary symptom flare. (DOCX 35 kb) [file 12894_2017_251_MOESM2_ESM.docx]

| **Additional file 2: Table S1. Time to urinary symptom flare** | | | |  |  |  |
| --- | --- | --- | --- | --- | --- | --- |
| **Time after implant** |  | **IPSS flare** | |  | **OABSS flare** | |
|  |  | **Increase of ≥ 6** | **Increase of ≥ 12** |  | **Increase of ≥ 3** | **Increase of ≥ 6** |
| Total |  | 183 (100%) | 83 (100%) |  | 189 (100%) | 78 (100%) |
| <1 year |  | 37 (20.2%) | 15 (18.1%) |  | 26 (13.8%) | 15 (10.3%) |
| 1 - 2 year |  | 40 (21.9%) | 18 (21.7%) |  | 37 (19.6%) | 18 (19.2%) |
| 2 - 3 year |  | 51 (27.9%) | 23 (27.7%) |  | 55 (29.1%) | 23 (32.1%) |
| 3 - 4 year |  | 25 (13.7%) | 9 (10.8%) |  | 34 (18.0%) | 9 (11.5%) |
| 4 - 5 year |  | 30 (16.4%) | 18 (21.7%) |  | 37 (19.6%) | 18 (26.9%) |
| IPSS = International prostate symptom score; OABSS = Overactive bladder symptom score | | | | | | |
