# Supplementary material for: Assessment of lower urinary symptom flare with overactive bladder symptom score and International Prostate Symptom Score in patients treated with iodine-125 implant brachytherapy: long-term follow-up experience at a single institute
Source: BMC Urol. 2017 Aug 14;17:62. doi: 10.1186/s12894-017-0251-1 (PMC5556596; doi:10.1186/s12894-017-0251-1)
Supplement: Supplementary file 3 — Comparison of clinicopathologic parameters by IPSS flare and OABSS flare in the 355 patients. (DOCX 45 kb) [file 12894_2017_251_MOESM3_ESM.docx]

| **Additional file 3: Table S2. Comparison of clinicopathologic parameters by IPSS flare and OABSS flare in the 355 patients** | | | | | | | | | | |
| --- | --- | --- | --- | --- | --- | --- | --- | --- | --- | --- |
| **Variables** |  | **Total (n = 355)** |  | **IPSS flare** | | |  | **OABSS flare** | | |
|  |  |  |  | **Non-flare (n=272)** | **Flare (n=83)** | **P value** |  | **Non-flare (n=277)** | **Flare (n=78)** | **P value** |
| **Age at brachyterapy (year)** |  | **71 (48 - 83)** |  | **70 (48 - 83)** | **71 (55 - 80)** | **0.45 ¶** |  | **70 (48 - 83)** | **72.5 (56 - 80)** | **0.06 ¶** |
| **Initial PSA (ng/mL)** |  | **7.11 (3.10 - 32.2)** |  | **7.18 (3.10 - 32.1)** | **6.96 (3.71 - 32.2)** | **0.20 ¶** |  | **7.19 (3.10 - 32.1)** | **6.92 (3.52 - 32.2)** | **0.60 ¶** |
| **Clinical T category** |  |  |  |  |  | **0.61 §** |  |  |  | **0.72 §** |
| **T1c** |  | **197 (55%)** |  | **148 (54%)** | **49 (59%)** |  |  | **154 (55%)** | **43 (55%)** |  |
| **T2a** |  | **128 (36%)** |  | **101 (37%)** | **27 (33%)** |  |  | **98 (36%)** | **30 (38%)** |  |
| **T2b/T2c/T3a** |  | **30 (8%)** |  | **23 (9%)** | **7 (8%)** |  |  | **25 (9%)** | **5 (7%)** |  |
| **D'Amico risk classification** |  |  |  |  |  | **0.15 §** |  |  |  | **0.78 §** |
| **Low** |  | **153 (43%)** |  | **124 (46%)** | **29 (35%)** |  |  | **118 (43%)** | **35 (45%)** |  |
| **Intermidiate** |  | **166 (47%)** |  | **124 (46%)** | **42 (50%)** |  |  | **132 (48%)** | **34 (44%)** |  |
| **High** |  | **36 (10%)** |  | **24 (8%)** | **12 (25%)** |  |  | **27 (9%)** | **9 (11%)** |  |
| **Gleason sum** |  |  |  |  |  | **0.09 §** |  |  |  | **0.60 §** |
| **6** |  | **206 (58%)** |  | **165 (61%)** | **41 (49%)** |  |  | **157 (57%)** | **49 (63%)** |  |
| **7** |  | **131 (37%)** |  | **96 (35%)** | **35 (42%)** |  |  | **106 (38%)** | **25 (32%)** |  |
| **8 or 9** |  | **18 (5%)** |  | **11 (4%)** | **7 (9%)** |  |  | **14 (5%)** | **4 (5%)** |  |
| **Hypertention** |  |  |  |  |  | **0.63 §** |  |  |  | **0.89 §** |
| **No** |  | **243 (68%)** |  | **188 (69%)** | **55 (66%)** |  |  | **189 (68%)** | **54 (69%)** |  |
| **Yes** |  | **112 (32%)** |  | **84 (31%)** | **28 (34%)** |  |  | **88 (32%)** | **24 (31%)** |  |
| **Diabetes mellitus** |  |  |  |  |  | **0.02 §** |  |  |  | **0.43 §** |
| **No** |  | **318 (90%)** |  | **238 (88%)** | **80 (96%)** |  |  | **250 (90%)** | **68 (85%)** |  |
| **Yes** |  | **37 (10%)** |  | **34 (12%)** | **3 (4%)** |  |  | **27 (10%)** | **10 (15%)** |  |
| **Pre-use of alpha-1 antagonist** |  |  |  |  |  | **0.98 §** |  |  |  | **0.31 §** |
| **No** |  | **304 (86%)** |  | **233 (85%)** | **71 (85%)** |  |  | **240 (86%)** | **64 (82%)** |  |
| **Yes** |  | **51 (14%)** |  | **39 (25%)** | **12 (15%)** |  |  | **37 (14%)** | **14 (18%)** |  |
| **Baseline IPSS** |  |  |  |  |  |  |  |  |  |  |
| **Continuous value** |  | **7 (0 - 33)** |  | **7 (0 - 33)** | **6 (0 - 24)** | **0.61 ¶** |  | **7 (0 - 33)** | **6 (0 - 29)** | **0.34 ¶** |
| **0 to 7** |  | **202 (57%)** |  | **154 (56%)** | **48 (58%)** | **0.78 §** |  | **152 (55%)** | **50 (64%)** | **0.32 §** |
| **8 to 19** |  | **130 (37%)** |  | **99 (37%)** | **31 (37%)** |  |  | **107 (39%)** | **23 (30%)** |  |
| **20 to 35** |  | **23 (7%)** |  | **19 (7%)** | **4 (5%)** |  |  | **18 (6%)** | **5 (6%)** |  |
| **Maximal IPSS after implant** |  | **20 (0 - 35)** |  | **19 (0 - 35)** | **22 (1 - 35)** | **0.12 ¶** |  | **20 (0 - 33)** | **21 (4 - 35)** | **0.74 ¶** |
| **Baseline OABSS** |  |  |  |  |  |  |  |  |  |  |
| **Continuous value** |  | **3 (0 - 13)** |  | **3 (0 - 13)** | **3 (0 - 13)** | **0.41 ¶** |  | **3 (0 - 13)** | **3 (0 - 13)** | **0.10 ¶** |
| **0 to 5** |  | **291 (82%)** |  | **222 (83%)** | **69 (82%)** | **0.84 §** |  | **231 (83%)** | **63 (81%)** | **0.39 §** |
| **6 to 11** |  | **59 (17%)** |  | **43 (16%)** | **16 (17%)** |  |  | **44 (16%)** | **13 (17%)** |  |
| **12 to 15** |  | **4 (1%)** |  | **3 (1%)** | **1 (1%)** |  |  | **2 (1%)** | **2 (2%)** |  |
| **Maximal OABSS after implant** |  | **7 (0 - 15)** |  | **7 (0 - 13)** | **8 (1 - 15)** | **0.24 ¶** |  | **7 (0 - 15)** | **8 (1 - 15)** | **0.10 ¶** |
| **Baseline toal score of IIEF-5** |  | **5 (1 - 25)** |  | **5 (1 - 25)** | **6 (1 - 25)** | **0.66 ¶** |  | **5 (1 - 25)** | **5 (1 - 25)** | **0.53 ¶** |
| **Prostate volume at diagnosis (mL)** |  | **24.2 (7.8 - 59.9)** |  | **24.2 (7.8 - 59.9)** | **24.5 (9.5 - 58.6)** | **0.40 ¶** |  | **24.1 (7.8 - 59.9)** | **25.1 (11.8 - 58.6)** | **0.42 ¶** |
| **Prostate volume at implant (mL)** |  | **25.7 (7.8 - 61.9)** |  | **25.7 (7.8 - 55.2)** | **25.7 (10.5 - 61.9)** | **0.40 ¶** |  | **25.2 (7.8 - 55.2)** | **27.0 (12.5 - 61.9)** | **0.22 ¶** |
| **Treatment parameters** |  |  |  |  |  |  |  |  |  |  |
| **Supplementary EBRT** |  |  |  |  |  | **0.83 §** |  |  |  | **0.94 §** |
| **No** |  | **247** |  | **190 (70%)** | **57 (68%)** |  |  | **193 (70%)** | **54 (69%)** |  |
| **Yes** |  | **108** |  | **82 (30%)** | **26 (32%)** |  |  | **84 (30%)** | **24 (31%)** |  |
| **Combined ADT** |  |  |  |  |  | **0.16 §** |  |  |  | **0.76 §** |
| **No** |  | **227** |  | **168 (62%)** | **59 (71%)** |  |  | **176 (64%)** | **51 (65%)** |  |
| **Yes** |  | **128** |  | **104 (38%)** | **24 (29%)** |  |  | **101 (36%)** | **27 (35%)** |  |
| **No of needles** |  | **23 (14 - 36)** |  | **23 (14 - 36)** | **22 (16 - 30)** | **0.15 ¶** |  | **22 (14 - 36)** | **23 (16 - 30)** | **0.46 ¶** |
| **No of seeds** |  | **60 (30 - 95)** |  | **60 (30 - 95)** | **65 (35 - 90)** | **0.36 ¶** |  | **60 (30 - 95)** | **65 (35 - 90)** | **0.21 ¶** |
| **BED (Gy2)** |  | **194.8 (120.3 - 253.2)** |  | **192.7 (120.3 - 247.1)** | **194.8 (120.3 - 253.2)** | **0.02 ¶** |  | **193.8 (120.3 - 253.1)** | **199.4 (131.03 - 247.1)** | **0.39 ¶** |
| **All the continueous values are expressed as median and range; PSA = prostate-specific antigen; SD = standard deviation; EBRT = external beam radiotherapy; BED = biologically effective dose; IPSS = International Prostate Symptom Score; OABSS = overactive bladder symptom score. ¶, Comparison between non-flare cases and flare cases with Mann-Whitney U test; §, Comparison between non-flare cases and flare cases with chi-square test and Fisher’s exact test** | | | | | | | | | | |
|  |  |  |  |  |  |  |  |  |  |  |
