# Supplementary material for: Assessment of lower urinary symptom flare with overactive bladder symptom score and International Prostate Symptom Score in patients treated with iodine-125 implant brachytherapy: long-term follow-up experience at a single institute
Source: BMC Urol. 2017 Aug 14;17:62. doi: 10.1186/s12894-017-0251-1 (PMC5556596; doi:10.1186/s12894-017-0251-1)
Supplement: Supplementary file 4 — Characteristics of 247 patients without supplementary EBRT. (DOCX 37 kb) [file 12894_2017_251_MOESM4_ESM.docx]

| **Additional file 4: Table S3. Characteristics of 247 patients without supplementary EBRT** | | | |
| --- | --- | --- | --- |
| **Variables** |  | **Total (n = 247)** |  |
| **Age at brachyterapy (years) †** |  | **70 (48 - 83)** |  |
| **Initial PSA (ng/mL) †** |  | **6.50 (3.10 - 19.4)** |  |
| **Clinical T category** |  |  |  |
| **T1c** |  | **151** | **61%** |
| **T2a** |  | **91** | **37%** |
| **T2b/T2c** |  | **5** | **2%** |
| **D'Amico risk classification** |  |  |  |
| **Low** |  | **153** | **62%** |
| **Intermidiate** |  | **93** | **38%** |
| **High** |  | **1** | **0%** |
| **Gleason score** |  |  |  |
| **6** |  | **175** | **71%** |
| **7** |  | **72** | **29%** |
| **Hypertention** |  |  |  |
| **No** |  | **174** | **70%** |
| **Yes** |  | **73** | **30%** |
| **Diabetis** |  |  |  |
| **No** |  | **224** | **91%** |
| **Yes** |  | **23** | **9%** |
| **Pre-use of alpha-1 antagonist** |  |  |  |
| **No** |  | **211** | **85%** |
| **Yes** |  | **36** | **15%** |
| **Baseline IPSS (0 to 35)** |  |  |  |
| **Continuous value †** |  | **7 (0 - 33)** |  |
| **1 to 7** |  | **134** | **54%** |
| **8 to 19** |  | **95** | **38%** |
| **20 to 35** |  | **18** | **7%** |
| **Baseline OABSS (0 to 15)** |  |  |  |
| **Continuous value †** |  | **3 (0 - 13)** |  |
| **0 to 5** |  | **199** | **81%** |
| **6 to 11** |  | **46** | **19%** |
| **12 to 15** |  | **2** | **1%** |
| **Prostate volume at diagnosis (mL) †** |  | **24.5 (7.8 - 59.9)** |  |
| **Prostate volume at implant (mL) †** |  | **26.1 (7.8 - 55.2)** |  |
| **ADT** |  |  |  |
| **No** |  | **173** | **70%** |
| **Yes** |  | **74** | **30%** |
| **No of needles †** |  | **24 (15 - 36)** |  |
| **No of seeds †** |  | **65 (37 - 95)** |  |
| **Post-dosimetric parameters †** |  |  |  |
| **BED (Gy2)** |  | **179.8 (120.3 - 235.0)** |  |
| **D90 (Gy)** |  | **169.8 (115.78 - 218.5)** |  |
| **%D90 (%)** |  | **111.2 (79.8 - 136.6)** |  |
| **V100 (%)** |  | **94.9 (77.8 - 99.4)** |  |
| **V150 (%)** |  | **60.5 (26.43 - 82.5)** |  |
| **V200 (%)** |  | **28.7 (6.4 - 52.8)** |  |
| **UD30 (Gy)** |  | **208.9 (131.9- 296.9)** |  |
| **%UD30 (%)** |  | **137.6 (96.1 - 200.3)** |  |
| **UD90 (Gy)** |  | **147.1 (88.6 - 205.5)** |  |
| **%UD90 (%)** |  | **95.3 (61.1 - 141.7)** |  |
| **PSA = prostate-specific antigen; SD = standard deviation; EBRT = ; %D90: minimal percentage of the dose received by 90% of the prostate gland; D90: minimal does (Gy) received by 90% of the prostate gland; V100/V150/V200: percentage of the prostate volume receiving 100% and 150% of the prescribed minimal peripheral dose; %UD30/UD30: minimal percentage of the dose and minimal dose (Gy) received by 30% of the urethra); %UD90/UD90: minimal percentage of the dose and minimal dose (Gy) received by 90% of the urethra; BED: biologically effective dose; †, expressed by medians and ranges** | | | |
