# Supplementary material for: Assessment of lower urinary symptom flare with overactive bladder symptom score and International Prostate Symptom Score in patients treated with iodine-125 implant brachytherapy: long-term follow-up experience at a single institute
Source: BMC Urol. 2017 Aug 14;17:62. doi: 10.1186/s12894-017-0251-1 (PMC5556596; doi:10.1186/s12894-017-0251-1)
Supplement: Supplementary file 5 — Comparison by IPSS flare and OABSS flare in the 247 patients without supplementary EBRT. (DOCX 47 kb) [file 12894_2017_251_MOESM5_ESM.docx]

| **Additional file 5: Table S4. Comparison by IPSS flare and OABSS flare in the 247 patients without supplementary EBRT** | | | | | | | | | | |
| --- | --- | --- | --- | --- | --- | --- | --- | --- | --- | --- |
| **Variables** |  | **Total (n = 247)** |  | **IPSS flare** | | |  | **OABSS flare** | | |
|  |  |  |  | **Non-flare (n=190)** | **Flare (n=57)** | **P value** |  | **Non-flare (n=193)** | **Flare (n=54)** | **P value** |
| **Age at brachyterapy (year-old) †** |  | **70 (48 - 83)** |  | **69 (48 - 83)** | **71 (55 - 79)** | **0.95 ¶** |  | **69 (48 - 83)** | **72 (56 - 79)** | **0.95 ¶** |
| **Initial PSA (ng/mL) †** |  | **6.50 (3.10 - 19.4)** |  | **6.50 (3.10 - 18.0)** | **6.69 (3.71 - 19.4)** | **0.60 ¶** |  | **6.62 (3.10 - 18.0)** | **6.42 (3.52- 19.4)** | **0.55 ¶** |
| **Clinical T category** |  |  |  |  |  | **0.50 §** |  |  |  | **0.79 §** |
| **T1c** |  | **151 (61%)** |  | **114 (60%)** | **37 (65%)** |  |  | **120 (62%)** | **31 (57%)** |  |
| **T2a** |  | **91 (36%)** |  | **73 (38%)** | **18 (32%)** |  |  | **69 (35%)** | **22 (41%)** |  |
| **T2b/T2c** |  | **5 (2%)** |  | **3 (2%)** | **2 (3%)** |  |  | **4 (3%)** | **1 (2%)** |  |
| **D'Amico risk classification** |  |  |  |  |  | **0.14 §** |  |  |  | **0.62 §** |
| **Low** |  | **153 (62%)** |  | **124 (65%)** | **29 (51%)** |  |  | **118 (61%)** | **35 (65%)** |  |
| **Intermidiate** |  | **93 (38%)** |  | **65 (34%)** | **28 (49%)** |  |  | **75 (39%)** | **18 (33%)** |  |
| **High** |  | **1 (0%)** |  | **1 (1%)** | **0 (0%)** |  |  | **0 (0%)** | **1 (2%)** |  |
| **Gleason sum** |  |  |  |  |  | **0.15 §** |  |  |  | **0.80 §** |
| **6** |  | **175 (71%)** |  | **139 (73%)** | **36 (63%)** |  |  | **136 (70%)** | **39 (72%)** |  |
| **7** |  | **72 (29%)** |  | **51 (27%)** | **21 (37%)** |  |  | **57 (30%)** | **15 (28%)** |  |
| **Hypertention** |  |  |  |  |  | **0.47 §** |  |  |  | **0.99 §** |
| **No** |  | **174 (70%)** |  | **136 (72%)** | **38 (67%)** |  |  | **136 (70%)** | **38 (70%)** |  |
| **Yes** |  | **73 (30%)** |  | **54 (28%)** | **19 (33%)** |  |  | **57 (30%)** | **16 (30%)** |  |
| **Diabetes mellitus** |  |  |  |  |  | **0.23 §** |  |  |  | **0.60 §** |
| **No** |  | **224 (91%)** |  | **170 (89%)** | **54 (95%)** |  |  | **176 (91%)** | **48 (89%)** |  |
| **Yes** |  | **23 (9%)** |  | **20 (11%)** | **3 (5%)** |  |  | **17 (9%)** | **6 (11%)** |  |
| **Pre-use of alpha-1 antagonist** |  |  |  |  |  | **0.89 §** |  |  |  | **0.94 §** |
| **No** |  | **211 (85%)** |  | **162 (85%)** | **49 (86%)** |  |  | **118 (87%)** | **44 (82%)** |  |
| **Yes** |  | **36 (15%)** |  | **28 (15%)** | **8 (14%)** |  |  | **26 (13%)** | **10 (18%)** |  |
| **Baseline IPSS** |  |  |  |  |  |  |  |  |  |  |
| **Continuous value †** |  | **7 (0 - 33)** |  | **6.5 (0 - 33)** | **7 (0 - 24)** | **0.38** |  | **7 (0 - 33)** | **7 (0 - 29)** | **0.37 ¶** |
| **0 to 7** |  | **134 (54%)** |  | **103 (54%)** | **31 (54%)** | **0.46 §** |  | **102 (53%)** | **32 (59%)** | **0.67 §** |
| **8 to 19** |  | **95 (38%)** |  | **75 (39%)** | **24 (42%)** |  |  | **76 (39%)** | **19 (37%)** |  |
| **20 to 35** |  | **18 (7%)** |  | **16 (7%)** | **2 (4%)** |  |  | **15 (8%)** | **3 (4%)** |  |
| **Maximal IPSS after implant** |  | **21 (0 - 35)** |  | **20 (0 - 35)** | **23(4 - 34)** | **0.19 ¶** |  | **20 (0 - 35)** | **21.5 (4 - 35)** | **0.19 ¶** |
| **Baseline OABSS** |  |  |  |  |  |  |  |  |  |  |
| **Continuous value †** |  | **3 (0 - 13)** |  | **3 (0 - 12)** | **3 (0 - 13)** | **0.31 ¶** |  | **3 (0 - 11)** | **3 (0 - 13)** | **0.31 ¶** |
| **0 to 5** |  | **199 (80%)** |  | **154 (81%)** | **45 (79%)** | **0.72 §** |  | **156 (81%)** | **43 (80%)** | **0.84 §** |
| **6 to 11** |  | **46 (19%)** |  | **35 (18%)** | **11 (19%)** |  |  | **37 (19%)** | **9 (17%)** |  |
| **12 to 15** |  | **2 (1%)** |  | **1 (1%)** | **1 (2%)** |  |  | **0 (0%)** | **2 (3%)** |  |
| **Maximal OABSS after implant †** |  | **7 (0 - 15)** |  | **7 (0 - 14)** | **8 (1 - 15)** | **0.25 ¶** |  | **7 (0 - 15)** | **8 (1 - 14)** | **0.25 ¶** |
| **Baseline toal score of IIEF-5 †** |  | **5 (1 - 25)** |  | **5 (1 - 25)** | **7 (1 - 25)** | **0.19 ¶** |  | **6 (1 - 25)** | **5 (1 - 25)** | **0.19 ¶** |
| **Prostate volume at diagnosis (mL) †** | | **24.5 (7.8 - 59.9)** |  | **24.9 (7.8 - 59.9)** | **24.1 (9.5 - 58.6)** | **0.58 ¶** |  | **25.0 (7.8 - 59.9)** | **23.9 (11.8 - 58.6)** | **0.58 ¶** |
| **Prostate volume at implant (mL) †** |  | **26.1 (7.8 - 55.2)** |  | **26.4 (7.8 - 55.2)** | **26.0 (10.5 - 42.5)** | **0.43 ¶** |  | **26.1 (7.8 - 55.2)** | **26.9 (12.5 - 42.5)** | **0.43 ¶** |
| **Treatment parameters** |  |  |  |  |  |  |  |  |  |  |
| **Combined ADT** |  |  |  |  |  | **0.31 §** |  |  |  | **0.69 §** |
| **No** |  | **173** |  | **130 (68%)** | **43 (75%)** |  |  | **134 (69%)** | **39 (72)** |  |
| **Yes** |  | **74** |  | **60 (32%)** | **14 (25%)** |  |  | **59 (31%)** | **15 (28%)** |  |
| **No of needles †** |  | **24 (15 - 36)** |  | **24 (15 - 36)** | **23 (16 - 30)** | **0.10 ¶** |  | **24 (15 - 36)** | **24 (18 - 30)** | **0.10 ¶** |
| **No of seeds †** |  | **65 (37 - 95)** |  | **65 (37 - 95)** | **65 (40 - 90)** | **0.95 ¶** |  | **65 (37 - 95)** | **65 (45 - 90)** | **0.95 ¶** |
| **Post-dosimetric parameters †** |  |  |  |  |  |  |  |  |  |  |
| **BED (Gy2)** |  | **179.8 (120.3 - 235.0)** |  | **179.2 (120.3 - 228.7)** | **181.8 (131.0 - 235.0)** | **0.06 ¶** |  | **178.5 (120.3 - 229.3)** | **189.0 (148.13 - 235.0)** | **0.003¶** |
| **D90 (Gy)** |  | **169.8 (115.78 - 218.5)** |  | **168.7 (115.7 - 213.5)** | **178.0 (141.2 - 218.5)** | **0.06 ¶** |  | **169.3 (115.7 - 213.0)** | **171.6 (125.5 - 218.5)** | **0.001¶** |
| **%D90 (%)** |  | **111.2 (79.8 - 136.6)** |  | **110.7 (79.8 - 133.4)** | **113.6 (91.8 - 136.6)** | **0.21 ¶** |  | **111.2 (79.8 - 133.1)** | **110.5 (86.6- 136.6)** | **0.14 ¶** |
| **V100 (%)** |  | **94.9 (77.8 - 99.4)** |  | **94.8 (77.8 - 99.3)** | **96.1 (85.6 - 99.4)** | **0.08 ¶** |  | **94.9 (77.8 - 99.4)** | **95.2 (83.4 - 99.2)** | **0.16 ¶** |
| **V150 (%)** |  | **60.5 (26.43 - 82.5)** |  | **61.3 (26.4 - 85.2)** | **59.1 (35.3 - 80.6)** | **0.73 ¶** |  | **61.6 (26.4 - 82.5)** | **57.0 (38.1 - 80.5)** | **0.78 ¶** |
| **V200 (%)** |  | **28.7 (6.4 - 52.8)** |  | **28.7 (6.4 - 52.8)** | **28.6 (9.0 - 50.1)** | **0.84 ¶** |  | **28.9 (6.4 - 52.8)** | **26.7 (16.6 - 50.1)** | **0.85 ¶** |
| **UD30 (Gy)** |  | **208.9 (131.9- 296.9)** |  | **209.0 (131.9 - 296.9)** | **207.6 (170.4 - 276.6)** | **0.36 ¶** |  | **209.1 (131.9 - 296.9)** | **206.6 (152.67 - 262.5)** | **0.34 ¶** |
| **%UD30 (%)** |  | **137.6 (96.1 - 200.3)** |  | **137.6 (96.1 - 200.3)** | **132.0 (106.5 - 172.9)** | **0.68 ¶** |  | **137.7 (96.1 - 200.3)** | **134.9 (103.8 - 164.3)** | **0.30 ¶** |
| **UD90 (Gy)** |  | **147.1 (88.6 - 205.5)** |  | **147.1 (88.6 - 205.5)** | **146.6 (107.2 - 193.7)** | **0.72 ¶** |  | **147.0 (101.6 - 181.5)** | **147.8 (101.6 - 141.7)** | **0.68 ¶** |
| **%UD90 (%)** |  | **95.3 (61.1 - 141.7)** |  | **96.0 (61.1 - 141.7)** | **94.7 (66.9 - 121.7)** | **0.37 ¶** |  | **96.0 (61.1 - 141.7)** | **94.1 (70.1 - 125.2)** | **0.32 ¶** |
| **All the continueous values are expressed by median and range; PSA = prostate-specific antigen; SD = standard deviation; EBRT = external beam radiotherapy; %D90 = minimal percentage of the dose received by 90% of the prostate gland; D90 = minimal does (Gy) received by 90% of the prostate gland; V100/V150 = percentage of the prostate volume receiving 100% and 150% of the prescribed minimal peripheral dose; %UD30/UD30 = minimal percentage of the dose and minimal dose (Gy) received by 30% of the urethra); %UD90/UD90 = minimal percentage of the dose and minimal dose (Gy) received by 90% of the urethra; BED = biologically effective dose; †, expressed by medians and ranges; ¶, Comparison between non-flare cases and flare cases with Mann-Whitney U test; §, Comparison between non-flare cases and flare cases with chi-square test and Fisher’s exact test** | | | | | | | | | | |
