# Supplementary material for: Assessment of lower urinary symptom flare with overactive bladder symptom score and International Prostate Symptom Score in patients treated with iodine-125 implant brachytherapy: long-term follow-up experience at a single institute
Source: BMC Urol. 2017 Aug 14;17:62. doi: 10.1186/s12894-017-0251-1 (PMC5556596; doi:10.1186/s12894-017-0251-1)
Supplement: Supplementary file 6 — Comparison of PSA bounce and urinary symptom flare in patients without androgen deprivation therapy. PSA bounce was defined as an elevation of ≥0.1 ng/mL compared to the previous lowest value, followed by a decrease to a level at or below the pre-bounce value. (DOCX 35 kb) [file 12894_2017_251_MOESM6_ESM.docx]

| **Additional file 6: Table S5. Comparison of PSA bounce and urinary symptom flare in patients without androgen deprivation therapy** | | | | | | | | | | |
| --- | --- | --- | --- | --- | --- | --- | --- | --- | --- | --- |
| **PSA bounce (≥ 0.1 ng/mL)** |  | **Total** |  | **IPSS flare** | | |  | **OABSS flare** | | |
|  |  |  |  | **Non-flare** | **Flare** | **P value** |  | **Non-flare** | **Flare** | **P value** |
| **Total** |  | **227** |  | **168** | **59** |  |  | **176** | **51** |  |
| **No** |  | **126** |  | **94 (74.6%)** | **32 (25.4%)** | **0.47 §** |  | **94 (74.6%)** | **32 (25.4%)** | **0.15 §** |
| **Yes** |  | **101** |  | **74 (73.3%)** | **27 (26.7%)** |  |  | **82 (81.2%)** | **19 (18.8%)** |  |
| **IPSS = International prostate symptom score; OABSS = Overactive bladder symptom score; §, Comparison between non-flare cases and flare cases with chi-square test** | | | | | | | | | | |
