# Supplementary material for: Assessment of lower urinary symptom flare with overactive bladder symptom score and International Prostate Symptom Score in patients treated with iodine-125 implant brachytherapy: long-term follow-up experience at a single institute
Source: BMC Urol. 2017 Aug 14;17:62. doi: 10.1186/s12894-017-0251-1 (PMC5556596; doi:10.1186/s12894-017-0251-1)
Supplement: Supplementary file 7 — Comparison of PSA bounce and urinary symptom flare in patients without androgen deprivation therapy. PSA bounce was defined as an elevation of ≥0.4 ng/mL compared to the previous lowest value, followed by a decrease to a level at or below the pre-bounce value. (DOCX 35 kb) [file 12894_2017_251_MOESM7_ESM.docx]

| **Additional file 7: Table S6. Comparison of PSA bounce and urinary symptom flare in patients without androgen deprivation therapy** | | | | | | | | | | |
| --- | --- | --- | --- | --- | --- | --- | --- | --- | --- | --- |
| **PSA bounce (≥ 0.4 ng/mL)** |  | **Total** |  | **IPSS flare** | | |  | **OABSS flare** | | |
|  |  |  |  | **Non-flare** | **Flare** | **P value** |  | **Non-flare** | **Flare** | **P value** |
| Total |  | 227 |  | 168 | 59 |  |  | 176 | 51 |  |
| No |  | 198 |  | 143 (83.8%) | 55 (16.2%) | 0.38 |  | 150 (75.8%) | 48 (24.2%) | 0.14 |
| Yes |  | 29 |  | 25 (86.3%) | 4 (13.7%) |  |  | 26 (89.7%) | 3 (10.3%) |  |
| IPSS = International prostate symptom score; OABSS = Overactive bladder symptom score | | | | | | | | | | |
